# Genetic Parameters of Methane Emission, Feed Efficiency, Feeding Behaviour, and Growth Traits in Beef Cattle

**DOI:** 10.1111/jbg.70037

**Published:** 2025-12-12

**Authors:** J. A. Silva, J. P. S. Valente, L. F. M. Mota, G. R. D. Rodrigues, T. L. S. Soares, J. O. S. Marcatto, A. M. Pelaez, F. M. Monteiro, R. C. Canesin, L. G. Albuquerque, M. E. Z. Mercadante

**Affiliations:** ^1^ Beef Cattle Research Center Institute of Animal Science Sertãozinho São Paulo Brazil; ^2^ School of Agriculture and Veterinary Science São Paulo State University Jaboticabal São Paulo Brazil; ^3^ Embrapa Environment Jaguariúna São Paulo Brazil; ^4^ Cia. de Melhoramento (Genetic Breeding Company) São José do Rio Preto São Paulo Brazil

**Keywords:** *Bos taurus indicus*, enteric methane, genetic correlation, heritability estimates

## Abstract

Climate change has intensified the need to reduce greenhouse gas emissions, particularly methane (CH_4_) from enteric fermentation. Genetic selection has emerged as a promising mitigation strategy; however, studies on *Bos taurus indicus* , especially Nellore cattle, remain limited. This study aimed to estimate heritabilities and genetic correlations for CH_4_ emission traits and their relationships with feeding behaviour, feed efficiency, and performance, as well as to evaluate the direct and correlated responses to selection for lower CH_4_ emissions. Data were from 2418 Nellore cattle evaluated in feed efficiency trials. Traits included dry matter intake (DMI), feeding time per day (FTd), feed events per day (FEd), and feeding rate (FR), residual feed intake (RFI), average daily gain (ADG), and mid‐test body weight (MBW). Methane emissions were measured in 1153 animals using the SF_6_ tracer technique, providing daily CH_4_ emission (g/day), CH_4_ per unit of DMI (CH_4_DMI, g/day), and residual CH_4_ (CH_4_res). Variance components were estimated using the single‐step genomic BLUP (ssGBLUP) method through Bayesian inference. Heritability estimates were moderate for CH_4_ (0.25), CH_4_DMI (0.14), CH_4_res (0.14), and performance traits such as DMI (0.35), ADG (0.36), and MBW (0.40). Higher estimates were observed for feeding behaviour traits FTd (0.49) and FR (0.42). Genetic correlations between CH_4_ and production traits were high, particularly with DMI (0.79), ADG (0.90), and MBW (0.91), indicating that selection for reduced CH_4_ emissions may affect growth. Direct selection for CH_4_ led to a modest annual reduction in emissions but also a correlated decline in MBW. These results demonstrate that while CH_4_ emissions are heritable, their strong genetic association with productivity traits indicates that isolated selection for reduced emissions may lead to undesirable outcomes in feed intake and performance. Therefore, strategies aiming to reduce CH_4_ emissions should consider the genetic relationships with growth and efficiency traits to avoid compromising animal productivity.

## 1 Introduction

Livestock systems face increasing pressure to improve production efficiency while meeting social and environmental demands, particularly in reducing greenhouse gas emissions (Cusack et al. 2021; Hyland et al. 2016; Min et al. 2022). In beef cattle, this challenge is critical, as ruminants are responsible for about 6% of global anthropogenic methane (CH_4_) emissions, a gas with a much higher global warming potential than carbon dioxide (CO_2_) (Pinto et al. 2022). Most CH_4_ is produced during microbial fermentation in the rumen, where microorganisms degrade feed, generating gases that are released mainly via belching (Berchielli et al. 2012; Choudhury et al. 2015; Cottle et al. 2011).

Combining genetic selection (González‐Recio et al. 2020; Hegarty et al. 2007) with nutritional strategies (Roque et al. 2019) is key to reducing CH_4_ emissions while maintaining efficiency. From a genetic standpoint, selection based on residual feed intake (RFI) is promising, as animals with negative RFI consume less feed than expected for their production level, thus emitting less CH_4_ overall (Fitzsimons et al. 2013; Sakamoto et al. 2021). In addition, direct selection for low CH_4_‐emitting animals is feasible due to the moderate heritability of the trait (Souza et al. 2024).

Although several studies have evaluated genetic parameters for CH_4_ emissions in cattle (de Haas et al. 2021; Hegarty et al. 2007; Holder et al. 2021; Min et al. 2022; Panwar et al. 2024), the genetic correlations between CH_4_ emissions and feed efficiency, feeding behaviour, and growth traits in beef cattle under tropical conditions remain unexplored. This knowledge gap is mainly due to the difficulty and high cost of obtaining large‐scale phenotypic data for these traits, especially CH_4_ emissions and feed intake behaviour, which require specialised equipment (Chipondoro 2024). Few studies have estimated genetic parameters for CH_4_ emissions alongside other economically relevant traits. For instance, Sepulveda et al. (2022) reported no genetic correlation between CH_4_ production and RFI in sheep but moderate correlations with feeding time, suggesting that behaviour may influence CH_4_ output.

Understanding genetic variation in CH_4_ emissions and its relationship with feeding behaviour, efficiency, and performance in Nellore cattle is essential for designing breeding programs that combine productivity with environmental sustainability (Johnson et al. 2022; Sepulveda et al. 2022). Therefore, this study aimed to estimate heritabilities and genetic and phenotypic correlations among CH_4_ emissions, feeding behaviour, feed efficiency, and performance traits and to assess the potential for selection to reduce CH_4_ emissions in Nellore cattle without compromising production.

## 2 Material and Methods

### 2.1 Ethical Approval

All phenotypic data and management procedures were collected following animal welfare guidelines, in accordance with State Law No. 11.977 of the State of São Paulo. The study was approved by the Ethics Committee of the Instituto de Zootecnia (Nova Odessa, SP, Brazil; Protocol No. 278‐19/2019).

### 2.2 Feed Efficiency Trials and Management

The dataset for CH_4_ emission, feeding behaviour, feed efficiency, and performance included male and female Nellore cattle born between 2004 and 2022 from three breeding programs: Institute of Animal Science (IZ; Sertãozinho, SP, Brazil), Cia. de Melhoramento (Fazenda Jacarezinho; Tangará da Serra, MT, Brazil), and Qualitas (Universidade Estadual Paulista; Botucatu, SP, Brazil).

Data on feeding behaviour, feed efficiency, and performance were measured during feed efficiency trials. CH_4_ emission was measured either during the trial or outside the trial period, depending on the breeding program protocol (see Table S1). The Institute of Animal Science performed multiple post‐weaning trials from 2005 to 2023, evaluating animals born between 2004 and 2022 (average age: 287 ± 42.72 days). In contrast, the other herds measured CH_4_ in yearling animals; Cia. de Melhoramento evaluated animals born in 2019 (average age: 542 ± 26.54 days), and Qualitas evaluated animals born in 2017 (average age: 657 ± 36.9 days). Differences in starting age reflect program‐specific trial design. Trial conditions for each breeding program are summarised in Table 1.

**TABLE 1:** Number of records per trait category and trial across three Nellore cattle breeding programs in Brazil.

Breeding program	Institute of Animal Science	Cia. de Melhoramento	Qualitas
Feed behaviour, feed efficiency and performance traits			
*N* of observations, *n*	2149	153	116
Birth, year	2004–2022	2018	2017
Trial duration^a^, ^b^, days	84 ± 12	76 ± 3	58 ± 0
Average daily gain, kg/day	1.1	1.1	1.7
Age at the beginning of the trial^b^, days	287 ± 42	542 ± 27	657 ± 37
Electronic feeder	Vytelle SENSE	Intergado	Intergado
Methane traits			
*N* of observations, *n*	866	153	111
Birth, year	2010, 2011, 2017–2022	2018	2017
Measurements, month	August–December	December–January	August
Methane measurements	Inside the trial	Outside the trial	Outside the trial

^a^ Minimum of 21 days for dietary and facilities adaptation.

^b^ Mean ± standard deviation (SD).

Diet formulations differed between breeding programs and years, reflecting the availability of feed resources and program objectives. Despite these differences, within each trial the diet composition and nutrient values, including neutral detergent fibre (NDF), acid detergent fibre (ADF), crude protein, dry matter, and gross energy, remained constant throughout the entire trial and methane measurement periods. Detailed ingredients and nutrient composition by trial and year are provided in Table S2. Feed was offered twice daily (08:00 and 15:00 h) with 5 to 10% orts allowed. Orts were removed three times a week before the morning feeding. All animals had *ad libitum* access to food, clean water, and mineral salt.

Feeding behaviour and individual feed intake were monitored using Vytelle SENSE (Vytelle, Kentucky, USA) or Intergado (Ponta, Betim, Minas Gerais, Brazil) electronic feeders, which restrict access to one animal at a time with containment bars. To be recognized by the system, each animal was equipped with a radiofrequency identification tag (RFID; Allflex, Joinville, SC, Brazil), enabling automatic identification. The system recorded individual ID, date and time of entry, feed intake (g), and feeding duration (s) for each feeding event.

Contemporary groups (CGs) for methane emission were defined as animals kept in the same pen, during the same trial, and fed the same diet, totaling 30 CGs. For feeding behavior, feed efficiency, and performance traits, CGs were defined by trial and diet only, regardless of pen, totaling 24 CGs.

#### 2.2.1 Measurement of Methane Emission

Emissions of CH_4_ were estimated using the sulfur hexafluoride (SF_6_) tracer gas technique described by Johnson and Johnson (1995) and Berndt et al. (2014). Briefly, each animal received an SF_6_ capsule inserted into the reticulum and underwent a 7‐day adaptation period to the equipment, including halters and sampling canisters. For CH_4_ measurement, animals were fitted with halters containing a filter, capillary tube, and vacuum reservoir. The inlet of the halter was positioned over the right nostril, and the outlet was connected to a stainless‐steel vacuum cylinder (2 L, −13.6 PSI) to collect eructated gases (CH_4_ and SF_6_). The capillary tube was calibrated to draw 50% of the vacuum volume over a 24‐h period.

The sampling cylinders were replaced daily for seven consecutive days, ensuring at least five valid samples per animal, corresponding to a total of 120 h of gas sampling per individual. Samples were analysed by gas chromatography at the EMBRAPA Meio Ambiente unit (Jaguariúna, SP, Brazil) to determine CH_4_ and SF_6_ concentrations in each cylinder. The amount of eructated CH_4_ was estimated based on the ratio of CH_4_ to SF_6_ concentrations in the samples, multiplied by the known release rate of the SF_6_ capsule. The release rate was determined by weekly weighing of each capsule for at least eight consecutive weeks prior to insertion into the animal's rumen (Sakamoto et al. 2021). Calculations were adjusted to account for background gas concentrations in the environment and the respective molecular weights of CH_4_ (16 g/mol) and SF_6_ (146 g/mol). Environmental sampling was performed simultaneously at two locations inside the pen, selected based on where animals spend most of their time. The same sampling system used on the animals was employed to quantify background concentrations and correct the CH_4_ estimates accordingly.

Individual daily CH_4_ emission (g/day) was calculated according to the following equation, adapted from Berndt et al. (2014):

$${\mathrm{ECH}}_{4}=\left[{\mathrm{ESF}}_{6}*\left(\frac{\left({\mathrm{CH}}_{4}C-{\mathrm{CH}}_{4}\mathrm{En}\right)}{{\mathrm{SF}}_{6}C-{\mathrm{SF}}_{6}\mathrm{En}}\right)*\left(\frac{\mathrm{MW}{\mathrm{CH}}_{4}}{\mathrm{MW}{\mathrm{SF}}_{6}}\right)\right]*1000$$

where ECH_4_ is the CH_4_ emission rate (g/day) in each cylinder; ESF_6_ is the emission rate of the SF_6_ capsule (mg/day); CH_4_C is the concentration of CH_4_ in the animal's cylinder (ppm); CH_4_En is the concentration of CH_4_ in the environment (ppm); SF_6_C is the concentration of SF_6_ in the animal's cylinder (ppt); SF_6_En is the concentration of SF_6_ in the environment (ppt); MWCH_4_ is the molecular weight of CH_4_ (16); MWSF_6_ is the molecular weight of SF_6_ (146), and 1000 is the conversion factor to express CH_4_ emission in each cylinder, in grams. CH_4_En and SF_6_En are the mean concentrations of the two cylinders placed in the pen.

The mean daily CH_4_ emission per animal was calculated as the arithmetic mean of five valid samples. Based on this value, two additional variables were derived: methane emission per kilogram of dry matter intake (CH_4_DMI, g/kg) and residual methane emission (CH_4_res, g/day). CH_4_res was defined as the residual of the linear regression between daily CH_4_ emission and DMI, as follows:

$${\mathrm{CH}}_{4}=\beta 0+\beta 1*\mathrm{DMI}+\varepsilon$$

where ${\mathrm{CH}}_{4}$ is daily emission; β0 is the intercept; β1 is the regression coefficient; DMI is the dry matter intake during the trial period, and ε is the residual of the equation that corresponds to CH_4_res.

#### 2.2.2 Feeding Behaviour Traits

The following traits were considered for the analysis of feeding behaviour: feeding time per day (FT_d_, min/day), obtained as the mean time the animal remained at the feeder per day of the feed efficiency trial; feed events per day (FE_d_, *n*/day), calculated as the average number of feed events per day during the feed efficiency trial; and feeding rate (FR, g/min), calculated as the ratio between total DMI and total FT_d_ during the feed efficiency trial.

#### 2.2.3 Feed Efficiency and Performance Traits

During the feed efficiency trial, the animals were weighed within a maximum interval of 28 days to determine the average daily gain (ADG) and mid‐trial body weight (MBW). The ADG was estimated by the linear regression coefficient (b_1_) of weights as a function of the days on trial (DOT), according to the following equation:

$$y_{i}=b_{0}+b_{1}*{\mathrm{DOT}}_{i}+e_{i}$$

where $y_{i}$ is the animal's weight in the *i*th observation; $b_{0}$ is the intercept that represents the initial weight at the beginning of the trial; $b_{1}$ is the linear regression coefficient representing ADG, and $e_{i}$ is the random error associated with each observation.

The MBW was calculated as follows:

$$\mathrm{MBW}=b_{0}+b_{1}\left(\frac{\mathrm{DOT}}{2}\right)$$

where $b_{0}$ is the weight at the beginning of the trial, and $b_{1}$ represents ADG.

Dry matter intake (DMI) was determined from the mean daily intake recorded during DOT using electronic feeders. The dry matter percentage was estimated from weekly samples of diet ingredients dried in an oven at 105°C to determine the dry matter content of the diet and thus to adjust the DMI.

The RFI was calculated as the residual of the multiple regression equation of DMI on ADG and mid‐trail metabolic body weight (BW^0.75^) within each trial group, as described by Koch et al. (1963):

$$\mathrm{DMI}=b_{0}+b_{1}*\mathrm{ADG}+b_{2}*{\mathrm{BW}}^{0.75}+e$$

where $b_{0}$ is the intercept; $b_{1}$ and $b_{2}$ are the linear regression coefficients for ADG and BW^0.75^, respectively, and e is the residual of the equation that represents the RFI.

### 2.3 Genotype and Pedigree Files

The animals were genotyped with the Illumina BovineHD BeadChip 770 K (Illumina INC., San Diego, CA, USA), the GeneSeek Genomic Profiler HDi 75 K (GeneSeek Inc., Lincoln, NE, USA), and the GeneSeek Genomic Profiler HDi 50 K (GeneSeek Inc., Lincoln, NE, USA). Animals genotyped with medium‐density panels (50 K and 75 K) were imputed to HD chip using the FImpute 3 software (Sargolzaei et al. 2014), considering a reference population of 6862 animals genotyped with the 770 K HD chip which were sampled from the three breeding programs: IZ, Cia de Melhoramento, and Qualitas. The imputation accuracy was evaluated by splitting the reference population into three folds, with markers masked to mimic the medium‐density panel and achieved an imputation accuracy of approximately 0.98. Genotype quality control was performed by keeping only autosomal SNP markers with a minor allele frequency (MAF) > 0.05, *p*‐value > 10^−5^ for Hardy–Weinberg equilibrium, and call rate > 90% for markers and samples. After quality control, 3687 animals with 383,343 SNP markers remained in the genomic dataset.

### 2.4 Heritability Estimates and Genetic Correlations

The variance and covariance components were estimated by Bayesian inference using the single‐step GBLUP method (ssGBLUP) (Misztal et al. 2009). Heritability estimates were obtained using a single‐trait animal model, as follows:

y=Xβ+Za+e

where *y* is the vector of observed traits (CH_4_, CH_4_DMI, CH_4_res, FE_d_, FT_d_, FR, DMI, RFI, ADG, and MBW); *β* is the vector of fixed effects (CG) and the linear and quadratic effect of the animal's age at the beginning of the trial as covariate; *X* is the incidence matrix of fixed effects relating *β*; *a* is the vector of the additive genetic effect assumed to be $a\sim N\left(0,H\right)$, where *H* is the relationship matrix that combines pedigree (*n* = 6132) and genomic (*n* = 3687) information, and $\sigma_{a}^{2}$ is the additive genetic variance; is the incidence matrix of random effects relating a, and e is the vector of residual effects, assumed to be $e\sim N\left(0,I\sigma_{e}^{2}\right)$, where *I* is an identity matrix and $\sigma_{e}^{2}$ is the residual variance.

Two‐trait analysis under an animal model was applied to estimate the (co)variance components and genetic correlations of CH_4_‐related traits with feeding behaviour, feed efficiency and performance traits, as well as between CH_4_ traits. The following model was used:

$$\left[\begin{array}{c} y_{1}\\ y_{2} \end{array}\right]=\left[\begin{array}{cc} X_{1} & 0\\ 0 & X_{2} \end{array}\right]\left[\begin{array}{c} b_{1}\\ b_{2} \end{array}\right]+\left[\begin{array}{cc} Z_{1} & 0\\ 0 & Z_{2} \end{array}\right]\left[\begin{array}{c} a_{1}\\ a_{2} \end{array}\right]+\left[\begin{array}{c} e_{1}\\ e_{2} \end{array}\right]$$

where $y_{1}$ and $y_{2}$ are the vectors of observation for traits 1 and 2, respectively; $b_{1}$ and $b_{2}$ are the fixed effects of contemporary group and covariates for traits 1 and 2; $a_{1}$ and $a_{2}$ are the vectors of additive genetic effects, and $e_{1}$ and $e_{2}$ are the vector of residual effects. The incidence matrices *X* and *Z* relate *y* to the fixed effects (*b*) and random effects (*a*), respectively. The random effects of the animals and residuals were assumed to be normally distributed, as $a\sim \mathrm{MVN}\left(0,H⨂S_{a}\right)$ and $a\sim \mathrm{MVN}\left(0,I⨂S_{e}\right)$, where $S_{a}=\left[\begin{array}{cc} \sigma_{a1}^{2} & \sigma_{a1,2}\\ \sigma_{a1,2} & \sigma_{a2}^{2} \end{array}\right]$ is the genetic (co)variance matrix between traits and $S_{e}=\left[\begin{array}{cc} \sigma_{e1}^{2} & \sigma_{e1,2}\\ \sigma_{e1,2} & \sigma_{e2}^{2} \end{array}\right]$ is the residual (co)variance matrix.

In ssGBLUP, the pedigree‐based relationship matrix *A* ^−1^ is replaced with the pedigree‐genomic relationship matrix *H* ^−1^, as described by Aguilar et al. (2010) and Christensen and Lund (2010).

$$H^{-1}=A^{-1}+\left[\begin{array}{cc} 0 & 0\\ 0 & G^{-1}-A_{22}^{-1} \end{array}\right]$$

where *H* ^−1^ is the inverse of the relationship matrix that combines the relationship derived from pedigree and genomic information; $A_{22}^{-1}$ is the inverse of the relationship matrix for genotyped animals, and $G^{-1}$ is the inverse of the genomic relationship matrix described by van Raden (2008):

$$G=\frac{{\mathrm{MM}}^{\prime }}{2\sum p_{i}\left(1-p_{i}\right)}$$

where *M* is the matrix of SNP markers with codes 0, 1, and 2 for genotypes AA, AB, and BB, respectively, adjusted for allele frequency expressed as 2*p*_i_, with *p*_i_ representing the MAF. The G matrix was scaled based on A_22_ considering the default parameters of the pregsf90 v. 1.24 software (Misztal et al. 2021), as mean(diag(G)) = mean(diag(A_22_)), mean(offdiag(G)) = mean(offdiag(A_22_)) (Christensen and Lund 2010). Before performing the analyses, the pedigree was corrected based on the genotypes using the seekparentf90 software v. 1.46 (Misztal et al. 2021). The pedigree was pruned according to the default of the renumf90 software v. 1.158 (Misztal et al. 2021), which considers a three generations depth. The inbreeding coefficients and the polygenic effect were considered to perform the analyses.

The posterior distributions of the genetic parameters were estimated by Bayesian inference using the GIBBSF90+, v. 3.16, and POSTGIBBSF90, v.3.15, software (Misztal et al. 2021). Gibbs sampling consisted of 500,000 iterations, with a burn‐in of 100,000 and sampling every 5 cycles, resulting in 80,000 samples for the estimation of (co)variance components.

### 2.5 Calculation of Direct and Correlated Responses to Selection

The direct response to selection was estimated for the CH_4_ emission traits according to the equation proposed by Falconer and Mackay (1996):

$$∆G_{x}=\frac{R_{\mathrm{tix}}i_{x}\sigma_{\mathrm{ax}}}{\mathrm{GI}}$$

where $∆G_{x}$ is the annual genetic gain for CH_4_ traits; $R_{\mathrm{tix}}$ is the accuracy of genetic prediction for CH_4_ traits (assumed as the square root of heritability under direct selection); $i_{x}$ is the selection intensity, considering 7% selected males and 0% selected females (value: 1.92); $\sigma_{\mathrm{ax}}$ is the additive genetic standard deviation for CH_4_ traits, and GI is the generation interval, assumed to be 10 years (Benfica et al. 2020).

The correlated responses in FT_d_, FE_d_, FR, DMI, RFI, ADG, and MBW were calculated considering direct selection for CH_4_ using the following equation (Falconer and Mackay 1996):

$$∆G_{x/y}=\frac{r_{\mathrm{gyx}}R_{\mathrm{tiy}}i_{y}\sigma_{\mathrm{ax}}}{\mathrm{GI}}$$

where $∆G_{\frac{x}{y}}$ is the annual genetic gain in trait *x* due to selection on trait *y*; $r_{\mathrm{gyx}}$ is the genetic correlation between traits *y* and *x*; $R_{\mathrm{tiy}}$ is the accuracy of selection for trait *y*; $i_{y}$ is the selection intensity applied to trait *y*; $\sigma_{\mathrm{ax}}$ is the additive genetic standard deviation of trait *x*, and GI is the generation interval.

## 3 Results

### 3.1 Descriptive Statistics

Descriptive statistics for all traits are presented in Table 2. CH_4_ emission (g/day) showed considerable variability among animals, with values ranging from 54.67 to 444.44 g/day. Similarly, CH_4_DMI and CH_4_res exhibited substantial dispersion. Feeding behaviour traits (FT_d_, FE_d_, and FR) also showed moderate variation. As expected, RFI and CH_4_res had mean values close to zero by construction. Performance traits such as ADG and DMI showed wide ranges, reflecting differences across animals and breeding programs.

**TABLE 2:** Structure of the database and descriptive statistics including mean, standard deviation, minimum, maximum and coefficient of variation of methane emission, feeding behaviour, feed efficiency and performance traits of Nellore cattle.

Trait	No. of observations	Mean	SD	Minimum	Maximum	CV
Methane emission						
CH_4_ (g/day)	1130	174.8	62.63	54.67	444.44	0.36
CH_4_DMI (g/kg DMI)	1128	20.9	6.02	6.76	46.71	0.29
CH_4_res (g/day)	1129	0.0	28.10	−126.07	245.94	—
Feeding behaviour						
FT_d_ (min/day)	1552	94.7	26.51	28.70	213.10	0.28
FE_d_ (*n*/day)	1552	72.1	24.31	14.90	150.50	0.34
FR (g/min)	1552	93.2	25.37	32.40	284.80	0.27
Feed efficiency and performance						
DMI (kg/day)	2418	7.8	1.63	2.16	13.92	0.21
RFI (kg/day)	2416	0.0	0.66	−3.71	4.84	—
ADG (kg/day)	2416	1.1	0.38	0.06	3.34	0.34
MBW (kg)	2418	317.9	95.74	125.08	726.00	0.30

Abbreviations: ADG, average daily gain; CH_4_, daily methane emission; CH_4_DMI, methane emission per dry matter intake; CH_4_res, residual methane emission; CV, coefficient of variation; DMI, dry matter intake; FE_d_, feed events per day; FR, feeding rate; FT_d_, feeding time per day; MBW, mid‐trial body weight; RFI, residual feed intake; SD, standard deviation.

**FIGURE 1:**
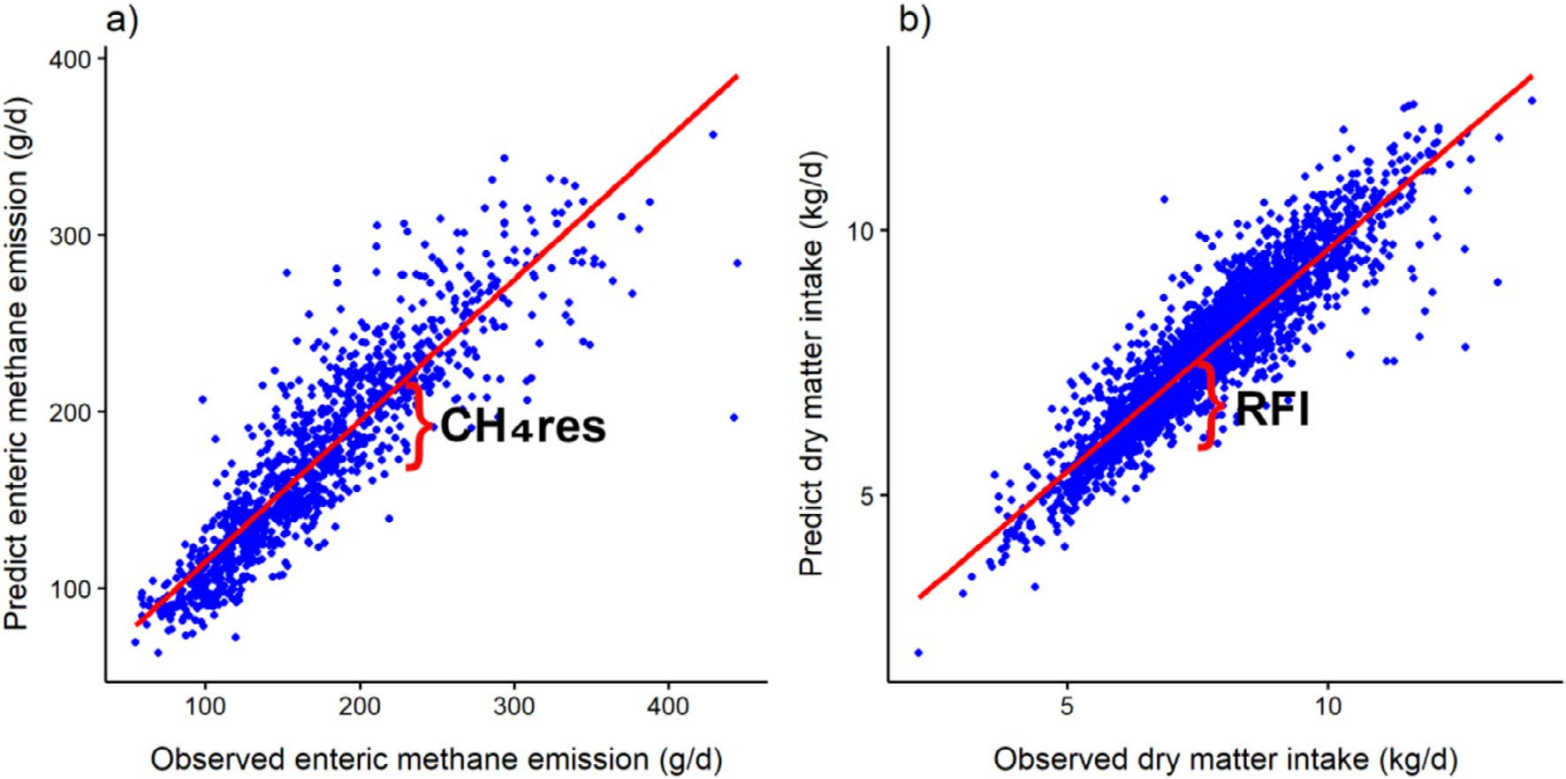
Relationship between observed and predicted values of methane emission and dry matter intake in Nellore cattle. (a) Residual methane emission (CH_4_res), calculated as the difference between observed enteric methane emission (g/day) and the value predicted by the linear regression model. (b) Residual feed intake (RFI), obtained as the difference between observed dry matter intake (kg/day) and the value predicted by the linear regression model. The coefficient of determination (*R* ^2^) was 0.63 for methane emission and 0.79 for dry matter intake. Brackets indicate the magnitude of residuals (CH_4_res or RFI), representing the portion of variation not explained by the model. The red line shows the fitted linear regression. [Colour figure can be viewed at wileyonlinelibrary.com]

The residual traits CH_4_res and RFI were obtained as the difference of observed and predicted values of methane emission (CH_4_) and dry matter intake (DMI), respectively (Figure 1). The coefficient of determination (*R* ^2^) was 0.63 for methane emission and 0.79 for DMI, indicating that the models explained a moderate to high proportion of the variation in the observed data. The residuals, highlighted in brackets, represent the deviation of each animal from the expected value based on its dry matter intake (CH_4_res) and on its performance and body weight (RFI) and reflect individual differences in methane emission and dry matter intake not accounted for by the model.

### 3.2 Heritability Estimates

The heritability estimates (*h* ^2^) and variance components for methane emission, feeding behaviour, feed efficiency, and performance traits are presented in Table 3. CH_4_ emission showed moderate heritability, while lower estimates were observed for CH_4_DMI and CH_4_res. Among feeding behaviour traits, FT_d_ showed the highest heritability, followed by FR and FE_d_. For feed efficiency and performance, heritability was moderate for DMI, ADG, and MBW, and RFI showed the lowest heritability within this group of traits.

**TABLE 3:** Additive genetic variance, residual variance, heritability ± SD, and 95% Highest Posterior Density interval for methane emission, feeding behaviour, feed efficiency, and performance traits in Nellore cattle.

Trait	$\sigma_{a}^{2}$	$\sigma_{e}^{2}$	*h* ^2^ ± SD	95% HPD	
				Lower	Upper
Methane emission					
CH_4_ (g/day)	275.5	810.5	0.25 ± 0.05	0.15	0.36
CH_4_DMI (g/kg DMI)	1.62	10.10	0.14 ± 0.05	0.04	0.24
CH_4_res (g/day)	111.2	699.2	0.14 ± 0.05	0.03	0.24
Feeding behaviour					
FTd (min/day)	272.1	278.5	0.49 ± 0.04	0.41	0.58
FEd (*n*/day)	62.3	128.1	0.33 ± 0.05	0.23	0.42
FR (g/min)	189.2	258.4	0.42 ± 0.05	0.33	0.52
Feed efficiency and performance					
DMI (kg/day)	0.32	0.60	0.35 ± 0.03	0.29	0.41
RFI (kg/day)	0.08	0.37	0.18 ± 0.03	0.12	0.24
ADG (kg/day)	0.02	0.03	0.36 ± 0.04	0.27	0.44
MBW (kg)	450.4	673.4	0.40 ± 0.04	0.33	0.47

Abbreviations: $\sigma_{a}^{2}$, additive genetic variance; $\sigma_{e}^{2}$, residual variance; ADG, average daily gain; CH_4_, daily methane emission; CH_4_DMI, methane emission per dry matter intake; CH_4_res, residual methane emission; DMI; dry matter intake; FE_d_, feed events per day; FR, feeding rate; FT_d_, feeding time per day; *h* ^2^, heritability; HPD, Highest Posterior Density interval; MBW, mid‐trial body weight; RFI, residual feed intake; SD, standard deviation.

### 3.3 Genetic and Phenotypic Correlations

The genetic correlations between methane emissions, feeding behaviour, feed efficiency, and performance traits in Nellore cattle are presented in Table 4. Daily CH_4_ emission showed a moderate and positive genetic correlation with CH_4_DMI (0.46 ± 0.19) and high and positive genetic correlation with CH_4_res (0.72 ± 0.10). Regarding feeding behaviour, CH_4_ emission was moderately and positively correlated with FT_d_, while FE_d_ and FR showed low genetic correlations with CH_4_. Among CH_4_ and the feed efficiency and performance traits, the genetic correlations with DMI, ADG, and MBW were high and positive. These associations indicate that animals with greater intake and better productive performance tend to emit more methane than less productive animals.

**TABLE 4:** Genetic correlations ± SD and 95% Highest Posterior Density (HPD) interval [lower; upper] between methane emission, feeding behaviour, feed efficiency and performance traits in Nellore cattle.

Trait	CH_4_DMI	CH_4_res	FT_d_	FE_d_	FR	DMI	RFI	ADG	MBW
CH_4_	0.46 ± 0.19 [0.10; 0.82]	0.72 ± 0.10 [0.52; 0.92]	0.28 ± 0.13 [0.03; 0.53]	0.03 ± 0.16 [−0.27; 0.34]	0.16 ± 0.13 [−0.10; 0.42]	0.79 ± 0.10 [0.59; 0.99]	0.27 ± 0.15 [−0.04; 0.57]	0.90 ± 0.09 [0.72; 1.08]	0.81 ± 0.10 [0.62; 1.01]
CH_4_DMI	—	0.90 ± 0.06 [0.79; 1.0]	−0.14 ± 0.17 [−0.47; 0.18]	0.02 ± 0.19 [−0.35; 0.38]	0.18 ± 0.18 [−0.18; 0.52]	−0.07 ± 0.15 [−0.36; 0.21]	−0.43 ± 0.21 [−0.85; −0.01]	0.24 ± 0.16 [−0.07; 0.55]	0.08 ± 0.14 [−0.18; 0.35]
CH_4_res	—	—	0.03 ± 0.17 [−0.31; 0.37]	0.02 ± 0.22 [−0.42; 0.45]	0.15 ± 0.19 [−0.22; 0.52]	0.19 ± 0.17 [−0.15; 0.53]	−0.07 ± 0.26 [−0.58; 0.44]	0.50 ± 0.21 [0.09; 0.90]	0.33 ± 0.17 [0.00; 0.66]

Abbreviations: ADG, average daily gain; CH_4_, daily methane emission; CH_4_DMI, methane emission per dry matter intake; CH_4_res, residual methane emission; DMI; dry matter intake; FE_d_, feed events per day; FR, feeding rate; FT_d_, feeding time per day; MBW, mid‐trial body weight; RFI, residual feed intake.

There was a strong positive genetic correlation between CH_4_DMI and CH_4_res. In contrast, correlations with most feeding behaviour and feed efficiency traits were low. As expected, the negative correlation between CH_4_DMI and RFI (−0.43 ± 0.21) suggests that more efficient animals tend to emit more methane per unit of DMI. For CH_4_res, low genetic correlations were observed with feeding behaviour and intake traits, while moderate correlations were found with ADG and MBW.

The phenotypic correlations between methane emission, feeding behaviour, feed efficiency, and performance traits in Nellore cattle are presented in Table 5. CH_4_ emission showed strong phenotypic correlations with CH_4_DMI and CH_4_res. The correlations between CH_4_ and feeding behaviour traits, as well as RFI, were weak. Moderate phenotypic correlations were observed between CH_4_ and DMI, ADG, and MBW. CH_4_DMI showed a strong phenotypic correlation with CH_4_ residual. Moderate correlations were observed with DMI and RFI. Correlations with feeding behaviour traits, ADG, and MBW were weak or close to zero. CH_4_res showed weak or near‐zero phenotypic correlations with feeding behaviour, DMI, RFI, ADG, and MBW.

**TABLE 5:** Phenotypic correlations ± SD and 95% Highest Posterior Density (HPD) interval [lower; upper] between methane emission, feeding behaviour, feed efficiency and performance traits of Nellore cattle.

Trait	CH_4_DMI	CH_4_res	FT_d_	FE_d_	FR	DMI	RFI	ADG	MBW
CH_4_	0.73 ± 0.02 [0.70; 0.76]	0.84 ± 0.01 [0.82; 0.86]	0.15 ± 0.03 [0.09; 0.22]	−0.02 ± 0.04 [−0.09; 0.05]	0.06 ± 0.03 [−0.01; 0.12]	0.35 ± 0.02 [0.30; 0.40]	0.10 ± 0.26 [0.04; 0.15]	0.26 ± 0.03 [0.21; 0.31]	0.38 ± 0.02 [0.33; 0.43]
CH_4_DMI		0.95 ± 0.00 [0.94; 0.96]	−0.13 ± 0.03 [−0.20; −0.06]	−0.14 ± 0.03 [−0.21; −0.07]	0.04 ± 0.03 [−0.02; 0.11]	−0.23 ± 0.03 [−0.28; −0.17]	−0.32 ± 0.03 [−0.37; −0.27]	−0.04 ± 0.03 [−0.10; 0.01]	0.03 ± 0.03 [−0.03; 0.08]
CH_4_res			−0.04 ± 0.03 [−0.11; 0.03]	−0.10 ± 0.04 [−0.17; −0.03]	0.03 ± 0.03 [−0.04; 0.09]	−0.07 ± 0.03 [−0.12; 0.01]	0.18 ± 0.03 [0.12; 0.24]	0.04 ± 0.03 [−0.01; 0.09]	0.12 ± 0.03 [0.06; 0.17]

Abbreviations: ADG, average daily gain; CH_4_, daily methane emission; CH_4_DMI, methane emission per dry matter intake; CH_4_res, residual methane emission; DMI; dry matter intake; FE_d_, feed events per day; FR, feeding rate; FT_d_, feeding time per day; MBW, mid‐trial body weight; RFI, residual feed intake.

### 3.4 Correlated Response to Selection

Table 6 shows the estimates of annual direct response estimates considering the selection of 7% of males based on the animal's own performance to selection for lower methane emission traits (CH_4_, CH_4_DMI and CH_4_res), and the correlated responses in feeding behaviour, feed efficiency, and performance traits. Selection for lower CH_4_ would result in a reduction in CH_4_ itself and in negative correlated responses in FT_d_, FR, and MBW. Correlated responses in FEd, FR, RFI, and ADG were close to zero. On the other hand, selection for lower CH_4_DMI resulted in a direct response of −0.09 g/kg DMI and negative correlated responses in FTd and FEd; however, there were no relevant effects on FR, DMI, RFI, ADG, or MBW, suggesting potential for reducing emissions without compromising performance.

**TABLE 6:** Estimates of the annual direct response to selection for lower methane emission traits and annual correlated responses in feeding behaviour, feed efficiency and performance in Nellore beef cattle.

Direct response		Correlated response						
		FT_d_ (min/day)	FE_d_ (*n*/day)	FR (g/min)	DMI (kg/day)	RFI (kg/day)	ADG (kg/day)	MBW (kg)
CH_4_ (g/day)	−1.60	−0.44	−0.03	−0.21	−0.04	−0.01	−0.01	−1.67
CH_4_DMI (g/kg DMI)	−0.09	−0.50	−0.25	−0.04	−0.01	0.00	0.00	−0.09
CH_4_res (g/day)	−0.75	−0.03	−0.01	−0.01	−0.01	0.00	0.00	−0.49

Abbreviations: ADG, average daily gain; CH_4_, daily methane emission; CH_4_DMI, methane emission per dry matter intake; CH_4_res, residual methane emission; DMI; dry matter intake; FE_d_, feed events per day; FR, feeding rate; FT_d_, feeding time per day; MBW, mid‐trial body weight; RFI, residual feed intake.

Selection for lower CH_4_res resulted in a direct response of −0.75 g/day and correlated responses of low magnitude in the other traits, with a reduction in MBW. These results indicate that, while selection based on CH_4_ can compromise feed intake and performance, the use of CH_4_DMI or CH_4_res can reduce methane emission with less impact on productive traits.

## 4 Discussion

Methane emissions from ruminants have gained increasing attention due to their contribution to greenhouse gas emissions and the global demand for more sustainable livestock systems (Gerber et al. 2013). Understanding the biological and genetic mechanisms related to methane production is important for developing mitigation strategies that do not compromise animal performance (de Haas et al. 2021; Knapp et al. 2014). In this study, genetic parameters for methane emissions and related traits were evaluated in Nellore cattle under feed efficiency trial conditions, using the SF_6_ tracer technique. The discussion below integrates the present findings with previous literature, considering the effects of diet, measurement methods, and trait heritabilities on methane emission patterns, and their associations with feeding behaviour, feed efficiency, and performance.

### 4.1 Descriptive Statistics

Methane emission estimates are strongly influenced by animal genetic composition, age, weight, diet, and measuring techniques In the present study, measurements were obtained during and after the feed efficiency trials, following each breeding program protocol, in which animals received diets formulated for an ADG of 1.1 (Animal Science Institute) or 1.7 kg/day (CIA de melhoramento and Qualitas), and methane was quantified using the SF_6_ tracer technique. Regarding measurement methods, few studies have reported CH_4_ emissions in beef cattle using the SF_6_ tracer. Using this technique, Maciel et al. (2019) reported CH_4_ emissions of 79.7 and 98.1 g/day under grazing conditions, and 168.7 and 209.8 g/day under feedlot conditions, for Nellore and Nellore × Angus cattle, respectively. Donoghue et al. (2016) reported mean values of 132.2 ± 25.4 g/day for CH_4_, 22.0 ± 2.3 g/kg for CH_4_DMI, and 0.0 ± 9.5 g/day for CH_4_res in 20‐month‐old Angus cattle using respirometry chambers. In Holstein cows evaluated with a non‐dispersive infrared technique, Manzanilla‐Pech et al. (2022) observed average CH_4_ emissions of 337.9 ± 86.2 g/day, 15.4 ± 3.4 g/kg for CH_4_DMI, and −0.1 ± 62.7 g/day for CH_4_res.

Some of the results reported in the literature were consistent with those obtained in the present study (Donoghue et al. 2016; Maciel et al. 2019), whereas others differ substantially (Manzanilla‐Pech et al. 2022). Such variation in CH_4_ emissions may be explained by physiological, metabolic, and nutritional factors linked to genetic composition (Berchielli et al. 2012; Knapp et al. 2014). Heavier and older animals generally exhibit higher dry‐matter intake, which increases CH_4_ production (Donoghue et al. 2016; Manzanilla‐Pech et al. 2022). Measurement technique is also an important source of variability; respirometry chambers capture all exhaled gases, the non‐dispersive infrared method quantifies concentrations in parts per million (ppm) later converted to g/day, while the SF_6_ tracer technique estimates emissions directly in g/day (Johnson and Johnson 1995). On the other hand, diet composition also plays a key role as fibre‐rich diets promote methanogenesis (Berchielli et al. 2012). In this study, the diet was formulated to achieve nutritional values similar to those of pasture‐fed animals, since most livestock in Brazil are raised on pasture. Consequently, high‐fibre diets tend to stimulate methane production by methanogenic bacteria compared with concentrate‐based diets. At the biochemical level, glucose derived from structural carbohydrates is broken down into two pyruvate molecules, precursors for volatile fatty acids (acetate, butyrate, and propionate). The metabolic pathway that leads to propionate formation does not generate methane. In contrast, during acetate production, one carbon atom is released, forming formate, which can be converted into methane. Similarly, in the formation of butyrate, two carbons are released, leading to the production of CO_2_ and CH_4_ in the animal's rumen.

The mean values for feeding behaviour, feeding efficiency, and performance in the present study suggest that the animals showed consistent feed intake patterns, reflecting adaptation to the management conditions. However, these averages should be interpreted considering the environment in which the trials were conducted. Group housing and a limited number of automatic feeders, may have increased competition among the animals, potentially influencing traits such as feeding time and number of visits. Thus, the observed values reflect not only intrinsic animal characteristics but also the influence of the management environment. Grant and Albright (2001), evaluating Nellore cattle at 13.5 ± 4.15 months of age in feed efficiency trials, reported estimates of 86.5 ± 21.5 min/day for FTd, 8.36 ± 2.0 kg/day for DMI, and 0.0 ± 0.71 kg/day for RFI, values close to those found in this study.

### 4.2 Heritability Estimates

The *h* ^2^ estimate for CH_4_ was of moderate magnitude, demonstrating its potential for genetic selection. The estimates for CH_4_DMI and CH_4_res were low, indicating that these traits are more affected by the environment. An acceptable explanation for the low *h* ^2^ estimates for CH_4_DMI and CH_4_res, compared to the *h* ^2^ for CH_4_, is the loss of a certain amount of additive genetic variation due to the correction for DMI. Manzanilla‐Pech et al. (2022) reported a similar *h* ^2^ estimate for CH_4_ emission (0.21 ± 0.05) in Holstein cattle measured with the non‐dispersive infrared technique. They also obtained higher *h* ^2^ estimates for CH_4_DMI and CH_4_res, 0.22 ± 0.05 and 0.16 ± 0.04, respectively. Likewise, Donoghue et al. (2016), evaluating Angus beef cattle with a portable accumulation chamber, reported *h* ^2^ estimates comparable to those observed in the present study of 0.27 ± 0.07 for CH_4_, 0.22 ± 0.06 for CH_4_DMI and 0.19 ± 0.05 for CH_4_res. Some divergence in estimates can be explained by differences in the sampling techniques of gas emission phenotypes (Johnson and Johnson 1995). Another factor that may influence the *h* ^2^ is the genetic composition of the herd. Donoghue et al. (2016) and Manzanilla‐Pech et al. (2022) evaluated taurine breeds whose metabolism and genes are different from those of the zebu animals used in this study.

In the present study, the *h* ^2^ estimates for feeding behaviour traits were of moderate to high magnitude, with the highest *h* ^2^ observed for FTd. A study evaluating the feeding behaviour of Holstein heifers reported similar *h* ^2^ estimates, with values of 0.50 ± 0.09, 0.45 ± 0.08, and 0.46 ± 0.09 for FTd, FEd, and FR, respectively (Lin et al. 2013). Another study assessing feeding behaviour in growing crossbred beef cattle found *h* ^2^ estimates of 0.25 ± 0.16 for FTd, 0.56 ± 0.19 for FEd, and 0.35 ± 0.16 for FR (Durunna et al. 2011). Despite the variation in these estimates, it is evident that feeding behaviour traits generally present moderate heritability, regardless of the environment in which the animals are evaluated, and may potentially be used as key traits for selection in the future.

The *h* ^2^ estimates for feed efficiency and performance traits in the present study were of moderate magnitude, except for RFI, which showed lower heritability. Previous studies, such as those by Donoghue et al. (2016), Johnson et al. (2022), and Sepulveda et al. (2022), reported higher *h* ^2^ estimates for DMI, ranging from 0.34 to 0.47 in taurine cattle and sheep. Lin et al. (2013) reported an *h* ^*2*^ estimate of 0.40 ± 0.09 for RFI in Holstein heifers. Studying Holstein heifers and cows, Freetly et al. (2020) estimated *h* ^2^ values of 0.25 ± 0.11 and 0.16 ± 0.10 for RFI, respectively. In sheep, Johnson et al. (2022) and Sepulveda et al. (2022) reported *h* ^2^ estimates of 0.42 ± 0.09 and 0.14 ± 0.04 for RFI, respectively. Heritability estimates for RFI vary across studies because it is a trait highly sensitive to environmental effects. This variation can be attributed to several biological factors such as feed intake regulation, digestive efficiency, metabolism (anabolism and catabolism), physical activity and thermoregulation (Herd et al. 2004). Richardson and Herd (2004) estimated that heat production from metabolic processes, body composition and activity together accounted for about 73% of the variation in RFI, distributed as protein turnover, tissue metabolism and stress (~37%); digestibility (~10%); heat increment and fermentation (~9%); physical activity (~9%); body composition (~5%); and feeding patterns (~2%). However, the remaining proportion of the variation could not be explained by these mechanisms, leaving gaps that make it difficult to fully account for variation in RFI and suggesting that other environmental effects not yet identified in the literature are also involved.

The *h* ^2^ estimates for ADG and MBW were of moderate magnitude. Performance traits commonly included in breeding programs are aimed at increasing weight gain and carcass yield. Donoghue et al. (2016) evaluated body weights at different ages and reported an *h* ^2^ of 0.46 ± 0.08 for yearling weight, an age comparable to that of the animals in the present study. In an analysis involving different cattle breeds, Berry and Crowley (2012) estimated *h* ^2^ values of 0.30 ± 0.06 for ADG and 0.69 ± 0.07 for metabolic body weight. Johnson et al. (2022) and Sepulveda et al. (2022) reported heritabilities of 0.42 ± 0.10 and 0.28 ± 0.05 for MBW, respectively. These findings indicate that weight‐related traits generally exhibit higher heritability and, consequently, greater potential for genetic improvement compared to traits such as RFI.

Heritability measures the strength of the relationship between phenotype and breeding value for a given trait within a population. While higher heritability generally suggests greater potential for genetic gain through selection, the actual gain also depends significantly on the breeding‐program structure and the amount of information available for each trait (Bourdon 2014). In the present study, several traits such as CH_4_, FTd, FEd, FR, DMI, ADG, and MBW, showed *h* ^2^ estimates of moderate to high magnitude, indicating their potential for inclusion in breeding objectives and the possibility of achieving genetic progress over generations. Notably, the *h* ^2^ for CH_4_ was moderate (0.25), which is promising for genetic selection. However, the existence of positive genetic correlations with productive traits such as DMI, ADG, and MBW may limit its isolated use, as selection for improved performance could lead to increased CH_4_ emissions through correlated responses.

### 4.3 Genetic and Phenotypic Correlations

The results of the present study indicate moderate to high genetic correlations (*r*_g_) among the methane emission‐related traits (CH_4_, CH_4_DMI, and CH_4_res), suggesting that these traits are influenced by the same genes through pleiotropic effects (Wagner and Zhang 2011). Therefore, selection to reduce any one of these CH_4_ emission traits is expected to result in correlated genetic responses in the others, since the mathematical relationships used to derive CH_4_DMI × CH_4_res from CH_4_ inherently generate covariances among them (Manzanilla‐Pech et al. 2022). Manzanilla‐Pech et al. (2022) and Sepulveda et al. (2022) estimated *r*_g_ values for CH_4_ × CH_4_DMI ranging from 0.77 ± 0.07 to 0.90 ± 0.14, and *r*_p_ values ranging from 0.54 ± 0.02 to 0.84 ± 0.01, demonstrating that higher daily emissions are associated with higher emissions per kg of DMI. Furthermore, the high *r*_g_ and *r*_p_ observed for CH_4_ × CH_4_res have been reported with values 0.74 ± 0.13 and 0.60 ± 0.02 (Sepulveda et al. 2022) and 0.72 ± 0.20 and 0.87 ± 0.01 (Richardson et al. 2021), respectively.

The *r*_g_ estimates between CH_4_ emission and feeding behaviour traits were mostly low, indicating a weak genetic relationship. However, the moderate *r*_g_ between CH_4_ × FTd suggests that animals spending more time at the feeder tend to emit more CH_4_. Benfica et al. (2020) reported *r*_g_ 0.54 ± 0.08 for FTd × DMI, supporting the idea that animals spending more time feeding tend to have higher intake and, consequently, higher methane emissions. Sepulveda et al. (2022) also found low genetic correlations, with estimates of 0.36 ± 0.19 for CH_4_ × FTd and −0.18 ± 0.27 for CH_4_ × FEd. The *r*_g_ values between CH_4_ × DMI, ADG, and MBW were of high magnitude, indicating a strong genetic association between these traits and CH_4_ emission. The genetic correlation between CH_4_DMI × RFI was moderate and negative, suggesting that animals with higher CH_4_ emission per kg of DMI tend to have lower RFI. Most traits of economic interest are positively correlated, meaning that selection for lower CH_4_ emissions may result in less productive animals as a correlated response. However, a selection index that includes multiple traits may help mitigate this issue, since DMI × MBW are genetically correlated with ADG (Benfica et al. 2020), one of the main breeding objectives focused on increasing productivity.

### 4.4 Direct and Correlated Responses to Selection

Breeding directly for daily CH_4_ emissions, as expected, will reduce CH_4_ emissions, but it will primarily affect feeding behaviour and MBW. In this approach, selecting for lower CH_4_ leads to EBVs for shorter daily time at the feeder and a lower FR, and the most adversely affected trait is MBW, whose EBV will decline each year (−1.67 kg/year). Ideally, selection should avoid changes in performance traits. This aligns with findings from de Haas et al. (2021), who showed that selection for less CH_4_ emission leads to decreases in feed intake traits and may slow genetic progress in performance traits, unless methane is assigned appropriate economic weight in selection indices. Consequently, although CH_4_‐focused selection is a powerful tool for mitigation, it presents production trade‐offs that may limit its adoption in commercial breeding programs.

Selection based on CH_4_DMI results in moderate reductions in emissions per kilogram of feed, with minimal or no adverse effects on animal performance. However, ratio traits like CH_4_DMI can be statistically and practically problematic. Improvements in the ratio may arise from changes in either the numerator (CH_4_) or denominator (DMI), making the genetic response less predictable and interpretation more complex. In addition, direct selection on ratio traits may unintentionally drive genetic gain in the wrong direction, as it is not controlled which of the underlying traits is being targeted. Zetouni et al. (2017), showed that multitrait selection using the component traits achieves higher and more predictable genetic gains for ratio traits compared to direct selection on the ratio trait. Therefore, caution should be taken when considering ratio traits as selection criteria.

Residual methane (CH_4_res) shows lower annual reductions in daily emissions compared to direct CH_4_ selection but is advantageous due to its independence from production traits. The findings indicate that CH_4_res is heritable and only weakly correlated with key performance traits, supporting its inclusion in multi‐trait selection indices aimed at reducing methane emissions without compromising productivity.

Overall, the results of this study provide a comprehensive overview of the genetic factors influencing methane emissions and their associations with feeding behaviour and key economically important traits in Nellore cattle. The moderate heritability of CH_4_ and its genetic correlations with intake and performance highlight the potential and challenges of isolated selection. In contrast, traits such as CH_4_DMI and CH_4_res offer alternative options for emission mitigation with fewer adverse effects on productivity. Nevertheless, it should be noted that ratio traits such as CH_4_DMI have their own statistical drawbacks, as discussed above. These findings reinforce the importance of multi‐trait selection indices that balance environmental goals with performance, ensuring feasible implementation in breeding programs (de Haas et al. 2021).

## 5 Conclusion

The genetic basis of methane emissions in Nellore cattle allows for the implementation of mitigation strategies through selective breeding. However, due to the genetic correlations between CH_4_ emissions and production traits, isolated selection for reduced emissions may lead to unfavorable outcomes in performance. Thus, sustainable genetic improvement requires a balanced selection approach that integrates methane reduction with feed efficiency and productivity. Future studies should evaluate the incorporation of CH_4_‐related traits into multi‐trait selection indices, which represent a feasible strategy to mitigate environmental impact without compromising production goals.

## Funding

The São Paulo Research Foundation (FAPESP) provided financial support (2017/10630‐2, 2017/50339‐5, 2021/11922‐2, and 2023/11428‐3) and scholarships to JPSV (2024/05697‐4), LFMM (2022/11852‐7), GRDR (2023/11176‐4), and TLSS (2023/14482‐9). The Coordination for the Improvement of Higher Education Personnel (CAPES) provided scholarships to JAS (Finance code 001). The National Council for Science and Technological Development (CNPq) (403115/2023‐0) provided financial support through competitive grants to LGA and MEZM.

## Ethics Statement

All phenotypic data and management procedures were collected following animal welfare guidelines, in accordance with State Law No. 11.977 of the State of São Paulo. The study was approved by the Ethics Committee of the Instituto de Zootecnia (Nova Odessa, SP, Brazil; Protocol No. 278‐19/2019).

## Conflicts of Interest

The authors declare no conflicts of interest.

## Supporting information


**Table S1:** Summary of trials, animals, and methane measurements using the SF_6_ tracer technique in three Nellore cattle breeding programs in Brazil.
**Table S2:** Composition and nutrient profile of diets provided during feed efficiency trials in three Nellore breeding programs in Brazil.

## Data Availability

The data were not deposited in an official repository. Data are available upon request to the corresponding author.
