# Supplementary material for: Genetic Parameters of Methane Emission, Feed Efficiency, Feeding Behaviour, and Growth Traits in Beef Cattle
Source: J Anim Breed Genet. 2025 Dec 12;143(4):496–507. doi: 10.1111/jbg.70037 (PMC13247634; doi:10.1111/jbg.70037)
Supplement: Supplementary file 1 — Table S1: Summary of trials, animals, and methane measurements using the SF6 tracer technique in three Nellore cattle breeding programs in Brazil. Table S2: Composition and nutrient profile of diets provided during feed efficiency trials in three Nellore breeding programs in Brazil. [file JBG-143-496-s001.docx]

**Supplementary Table S1.** Summary of trials, animals, and methane measurements using the SF₆ tracer technique in three Nellore cattle breeding programs in Brazil

| Breeding program | Animals birth (year) | Measurement (year) | Animals per group (n) | Feed efficiency period | Methane measured start date | Measurement phase  (inside trial / outside trial) |
| --- | --- | --- | --- | --- | --- | --- |
| Institute of Animal Science | 2010 | 2011 | 23 | Aug 3 to Oct 25 | Sept 13 | Inside |
|  |  |  | 23 | Jul 5 to Sept 14 | Sept 20 | Outside |
|  | 2011 | 2012 | 24 | Jul 12 to Oct 10 | Oct 23 |  |
|  |  |  | 25 | Aug 29 to Nov 22 | Dec 20 |  |
|  | 2017 | 2018 | 34 | Sept 25 to Dec 17 | Nov 26 | Inside |
|  |  |  | 36 | Sept 25 to Dec 17 | Dec 7 |  |
|  | 2018 | 2019 | 60 | Jun 18 to Sept 9 | Jun 24 |  |
|  |  |  | 62 | Sept 11 to Dec 3 | Oct 14 |  |
|  | 2019 | 2020 | 42 | Jun 17 to Sept 8 | Aug 5 |  |
|  |  |  | 44 | Jun 17 to Sept 8 | Aug 12 |  |
|  |  |  | 35 | Sept 9 to Nov 11 | Oct 6 |  |
|  |  |  | 34 | Sept 9 to Nov 11 | Oct 14 |  |
|  | 2020 | 2021 | 34 | Sept 9 to Nov 11 | Oct 14 |  |
|  |  |  | 34 | Sept 9 to Nov 17 | Oct 14 |  |
|  |  |  | 36 | Jun 22 to Sept 20 | Aug 2 |  |
|  |  |  | 39 | Jun 22 to Sept 20 | Aug 10 |  |
|  |  |  | 48 | Sept 21 to Dec 7 | Oct 18 |  |
|  |  |  | 48 | Sept 21 to Dec 7 | Oct 11 |  |
|  | 2021 | 2022 | 40 | Jun 21 to Sept 12 | Aug 1 |  |
|  |  |  | 38 | Jun 21 to Sept 12 | Aug 8 |  |
|  |  |  | 38 | Sept 14 to Nov 26 | Oct 3 |  |
|  |  |  | 35 | Sept 14 to Nov 26 | Oct 10 |  |
|  | 2022 | 2023 | 21 | Jun 20 to Sept 12 | Aug 15 |  |
|  |  |  | 23 | Jun 20 to Sept 12 | Aug 15 |  |
|  |  |  | 34 | Sept 12 to Nov 21 | Nov 15 |  |
|  |  |  | 30 | Sept 12 to Nov 21 | Nov 21 |  |
| Qualitas | 2017 | 2019 | 58 | Jul 3 to Aug 28 | Aug 13 |  |
|  |  |  | 58 | Jul 3 to Aug 28 | Aug 21 |  |
| Cia. de Melhoramento | 2020 | 2021 | 59 | Jan 12 to Mar 19 | Dec 13 | Outside |
|  | 2020 | 2022 | 45 | Jan 12 to Mar 19 | Feb 2 |  |
|  |  |  | 52 | Jan 12 to Mar 19 | Feb 7 |  |

| **Supplementary Table S2**. Composition and nutrient profile of diets provided during feed efficiency trials in three Nellore breeding programs in Brazil. | | | | | | | | | | |
| --- | --- | --- | --- | --- | --- | --- | --- | --- | --- | --- |
|  | IZ^1^ | IZ | IZ | IZ | Qualitas | IZ | IZ | IZ | Cia^2^ | IZ |
| Ingredient (%DM) | 2011 | 2012 | 2018 | 2019 | 2019 | 2020 | 2021 | 2022 | 2022 | 2023 |
| Corn silage | - | 53.6 | 54 | - | 27.6 | 60 | - | - | 82.9 | 60 |
| Sorgum silage | - | - | - | 60 | - | - | 60 | 60 | - | - |
| Bracharia grass | 44.5 | 10.1 | - | - | - | - | - | - | - | - |
| Sugar-cane bagasse | - | - | 10.2 | - | 4.89 | - | - | - | - | - |
| Cotton meal | 21.4 | - | - | - | - | - | - | - | - | - |
| Soybean meal | - | 11.6 | 11.7 | 13 | - | 13 | 13 | 13 | 14 | 13 |
| Peanut meal | - | - | - | - | 8.01 | - | - | - | - | - |
| Ground corn | 32.2 | 21.7 | 21.9 | 25 | - | 25 | 25 | 25 | - | 25 |
| Wet corn | - | - | - | - | 44.6 | - | - | - | - | - |
| Citrus pup | - | - | - | - | 11.9 | - | - | - | - | - |
| Mineral premix | - | - | - | - | 1.78 | - | - | - | 1.2 | - |
| Salt | 1.45 | 2.28 | 1.7 | 1.75 | - | 1.75 | 1.75 | 1.75 | 0.3 | 1.75 |
| Ammonium sulfate | - | 0.072 | - | - | - | - | - | - | - | - |
| Urea | 0.45 | 0.648 | 0.49 | 0.25 | 1.16 | 0.25 | 0.25 | 0.25 | 0.8 | 0.25 |
| Limestone | - | - | - | - | - | - | - | - | 0.8 | - |
| voluminous:concentrated | 65:35 | 65:35 | 60:40 | 60:40 | 50:50 | 60:40 | 60:40 | 60:40 | 82.9:17.1 | 60:40 |
| Nutrients |  |  |  |  |  |  |  |  |  |  |
| Dry matter % | 87.4 | 54.4 | 60.5 | 52.4 | 60 | 52.9 | 87.16 | 87.16 | 47.82 | 88.12 |
| Gross protein, %DM | 11.3 | 13.9 | 10.6 | 11.2 | 15.6 | 10.6 | 11.31 | 14.05 | 15.1 | 12.86 |
| Ashes, %DM | 3.75 | - | 3.69 | 4.63 | - | 4.08 | -7.04 | 3.88 | 5.92 | 4.67 |
| Neutral detergent fiber, %DM | 50 | 50.2 | 48.1 | 40.6 | 26.9 | 35.6 | 33.74 | 35.43 | 35.22 | 29.49 |
| Acid detergent fiber, %DM | 31 | 22.9 | 30.7 | 24.4 | - | 21.3 | 18.71 | 21.08 | 21.87 | 15.65 |
| Gross energy, Mcal/kg | 4.09 | 4.16 | 3.73 | 3.77 | 4.11 | 4.47 | 4.03 | 3.58 | - | 4.43 |
| Non-fibrous carbohydrates, %DM | 32.1 | 34 | 35.8 | 41.5 | 54 | 46.4 | - | - | 32.22 | - |
| Total digestible nutrients, %DM | 70.5 | 70.2 | 65.9 | 70.2 | 77 | 75.1 | - | - | - | - |

^1^IZ: Institute of Animal Science; ^2^Cia: Cia. de Melhoramento
